# Melanoma is associated with an increased risk of bullous pemphigoid: a large population-based longitudinal study

**DOI:** 10.1007/s00403-021-02211-4

**Published:** 2021-03-09

**Authors:** Khalaf Kridin, Jennifer E. Hundt, Ralf J. Ludwig, Kyle T. Amber, Dana Tzur Bitan, Arnon D. Cohen

**Affiliations:** 1 Lübeck Institute of Experimental Dermatology, University of Lübeck, Ratzeburger Allee 160, 23562 Lübeck, Germany; 2 Azrieli Faculty of Medicine, Bar-Ilan University, Safed, Israel; 3 Department of Dermatology, University of Illinois at Chicago, Chicago, IL USA; 4 Department of Behavioral Sciences, Ariel University, Ariel, Israel; 5 Clalit Health Services, Tel-Aviv, Israel; 6 Siaal Research Center for Family Medicine and Primary Care, Faculty of Health Sciences, Ben-Gurion University of the Negev, Beer Sheva, Israel

## Abstract

The association between bullous pemphigoid (BP) and melanoma is yet to be investigated. We aimed to assess assess the bidirectional association between BP and melanoma and to delineate the epidemiological features of patients with both diagnoses. A population-based cohort study was performed comparing BP patients (*n* = 3924) with age-, sex- and ethnicity-matched control subjects (*n* = 19,280) with regard to incident cases of melanoma. A case–control design was additionally adopted to estimate the risk of BP in individuals with a preexisting diagnosis of melanoma. The prevalence of preexisting melanoma was higher in patients with BP than in control subjects (1.5% vs. 1.0%, respectively; *P* = 0.004). A history of melanoma confers a 50% increase in the risk of subsequent BP (OR 1.53; 95% CI 1.14–2.06). This risk was higher among males (OR 1.66; 95% CI 1.09–2.54) and individuals older than 80 years (OR 1.63; 95% CI 1.11–2.38), and persisted after adjustment for multiple putative confounders including PD-1/PDL-1 antagonists (adjusted OR 1.53; 95% CI 1.14–2.06). Conversely, the risk of melanoma among patients with BP was slightly elevated, but did not reach the level of statistical significance (adjusted HR 1.13; 95% CI 0.73–1.74). Patients with a dual diagnosis of BP and melanoma were older at the onset of BP and had lower body mass index. A history of melanoma is associated with a 50% increase in the incidence of subsequent BP. Physicians managing patients with both conditions should be aware of this association. Further research is warranted to reveal the underlying mechanism of these findings.

**Supplementary Information:**

The online version contains supplementary material available at 10.1007/s00403-021-02211-4.

## Introduction

Bullous pemphigoid (BP) is the most prevalent subepidermal autoimmune bullous disease worldwide [1]. The disease is characterized by the presence of circulating IgG autoantibodies against BP180 and BP230, hemidesmosomal proteins promoting dermal-epidermal cohesion [2, 3]. Binding of these autoantibodies initiates blister formation via dermal-epidermal separation, which has been shown to be directly pathogenic [4]. The disease is clinically characterized by tense bullae, often in conjunction with urticarial plaques, and characteristically affects elderly individuals aged over 70 years. The last decades witnessed a considerable increase in the risk of the disease, which has been attributed, at least in part, to increased f longevity and exposure to several drugs [5]. The past few years yielded a prominent advance in elucidating the comorbidity profile of patients with BP owing to an increasing number of well-designed controlled observational studies [1, 6].

Melanoma is a malignant tumor that arises from melanocytes and is typified by a strong capacity to metastasize. While most commonly cutaneous in origin, it can potentially develop in mucosal surfaces, within the uveal tract, or in the leptomeninges [7]. Owing to its metastatic potential, melanoma accounts for more than 90% of skin cancer-related mortality [8]. The incidence of melanoma has risen substantially over the past four decades, whilst mortality rates follow a stable course since the early 1990s [9]. The prognosis of patients with melanoma varies in accordance with the stage at diagnosis. While patients with localized primary cutaneous melanoma have a favorable prognosis, the median survival time of patients with distant metastasis (before the advent of immunotherapies) was estimated at 9 months [7].

The coexistence of BP and melanoma was described in several case reports, but the association between these conditions is yet to be investigated by controlled observational studies [10]. The presence of a putative association between BP and melanoma was also postulated in view of several experimental and serological findings like the expression of BP180 in malignant melanocytes [11], the progression of melanoma in a mouse strain with BP180 dysfunction [12], and the increased seropositivity of anti-BP230 autoantibodies among melanoma patients [13].

The aim of the current study is to evaluate the association between BP and melanoma using a large-scale population-based study. We additionally aimed to examine whether patients with BP and melanoma possess a unique epidemiological profile relative to the remaining patients with BP.

## Methods

### Study design and dataset

The current study was performed to investigate the bidirectional association between BP and melanoma using one of the large cohorts of patients with BP in the literature. To delineate the risk of developing melanoma during the course of BP, a cohort study design was adopted to follow patients with BP and estimate the incidence of melanoma. To evaluate the risk of having BP in subjects with a preceding history of melanoma, a case–control study design was followed to unveil the prevalence of preexisting melanoma (exposure) in patients with subsequent BP (outcome). Given that ‘The rare disease assumption’ hypothesizes that estimates produced by case–control studies of rare disease approach that produced by cohort studies [14], the case–control study design allows us to assess the risk of BP in individuals with a history of melanoma.

The computerized dataset of Clalit Health Services (CHS) was the origin of the current study. CHS is the main healthcare maintenance organization in Israel, providing a wide array of private and public healthcare services for 4,927,000 enrollees as of October 2018. CHS retrieves data from a multitude of sources covering all strata of the healthcare system, including primary care healthcare facilities, referral centers settings, laboratory analyses, and imaging data. The loss to follow-up is negligible, and access to CHS services is free, thus increasing its compatibility to give rise to reliable epidemiological studies [15].

### Study population and definition of main variables

The dataset of CHS was systematically checked for incident cases with a diagnostic code BP between the years 2002 and 2019. Eventual eligibility in the study was only fulfilled when a patient met one of the following: (1) a documented diagnosis of BP registered at least twice by a board-certified dermatologist, or (2) a diagnosis of BP in discharge letters of patients admitted to dermatological wards. The diagnosis of melanoma was based on its documentation in the chronic registry of the CHS, which is cross-linked with the Israel National Cancer Registry. The control group was comprised of up to five individuals per each case of BP. Controls were matched based on sex, age, and ethnicity.

### Covariates and sensitivity analyses

To substantiate the validity of our findings, we performed two sensitivity analyses alongside the general analysis; (1) to increase the validity of the diagnosis of BP, only patients who claimed BP-related medications were included. The latter include systemic or topical corticosteroids for more than 6 months, as well as one of the adjuvant immunosuppressive or immunomodulatory agents (azathioprine, mycophenolate mofetil, methotrexate, cyclophosphamide, dapsone, doxycycline, rituximab, plasmapheresis, intravenous immunoglobulins); (2) to refute the presence of major ascertainment bias in the case–control study, we omitted cases of melanoma diagnosed up to 2 years prior to the study commencement.

Given the recent evidence connecting between the use of programmed death (PD)-1/programmed death ligand (PDL)-1 inhibitors in patients with metastatic melanoma and the development of BP [16], the outcome measures were additionally adjusted for this exposure. Since Parkinson disease was found to associate with both BP [17] and melanoma [18], we additionally adjusted for this variable as a putative confounder. To rule out ascertainment bias stemming from overutilization of healthcare services, and to ascertain that the study findings did not merely arise from over diagnosis of melanoma or BP, we additionally adjusted for utilization of healthcare services. This variable was defined as the number of visits to community physicians throughout the study duration (between 2002 and 2019).

### Statistical analysis

Baseline characteristics were described by means and standard deviations (SD)s for continuous variables, whilst categorical values were indicated by percentages. The comparison of different variables between cases and controls was performed using the chi-square test and *t* test for categorical and continuous variables, respectively.

In the cohort study design, incidence rates of melanoma were calculated for both BP patients and controls and expressed as the number of events per 1000 person-years. Hazard ratios (HR)s for the risk of incident melanoma were obtained by the use of the Cox regression model. In the case–control study design, logistic regression was used to calculate odds ratios (ORs) and 95% confidence intervals (CI)s to compare cases and controls with respect to the presence of preceding melanoma. The association was calculated based on individuals who developed BP after the diagnosis of melanoma, given that a temporal relationship exists between exposure and outcome in case–control studies. In the last section aiming to evaluate the epidemiological and clinical characteristics of patients with BP and melanoma relative to those with isolated BP, all patients with both diagnoses were included regardless of the sequence of their appearance. Two-tailed *P* values less than 0.05 were considered as statistically significant. All statistical analyses were performed using SPSS software, version 25 (SPSS, Armonk, NY: IBM Corp).

## Results

### Characteristics of the study population

A total of 3924 patients with BP and 19,280 age-, sex, and ethnicity-matched control subjects were included in the current study. The mean (SD) age at the onset of BP was 76.7 (14.3) years, 2257 (57.5%) were females, and 3752 (95.6%) were of Jewish ancestry. The basal characteristics of the study participants are outlined in Table 1.

**Table 1:** Descriptive characteristics of the study population

Characteristic	Patients with bullous pemphigoid (*N* = 3924)	Controls (*N* = 19,280)	*P* value
Age, years			
Mean (SD)	76.7 (14.3)	76.3 (14.3)	0.904
Median (range)	79.9 (0.4–104.4)	79.5 (0.7–103.8)	
Male sex, *N* (%)	1667 (42.5%)	8168 (42.4%)	0.908
Ethnicity, *N* (%)			
Jews	3752 (95.6%)	18,397 (95.4%)	0.584
Arabs	171 (4.4%)	868 (4.5%)	
BMI, mg/kg^2^			
Mean (SD)	27.9 (6.1)	27.9 (8.4)	1.000
Smoking, *N* (%)	1148 (29.3%)	5771 (29.9%)	0.454
Charlson comorbidity score			
Mean score (SD)	3.4 (2.4)	2.9 (2.3)	** < 0.001**
None (0)	468 (11.9%)	3376 (17.5%)	** < 0.001**
Moderate YY (1–2)	1113 (28.4%)	6177 (32.0%)	** < 0.001**
Severe (≥ 3)	2343 (59.7%)	9727 (50.5%)	** < 0.001**

*BP* bullous pemphigoid, *N* number, *SD* standard deviation, *BMI* body mass index

### The risk of developing BP with a preceding diagnosis of melanoma

The prevalence rate of preexisting melanoma was greater in patients with BP than in controls (1.5% vs. 1.0%, respectively; *P* = 0.004). To elaborate, a 1.5-fold increase in the risk of BP emerged in those with a history of melanoma (OR 1.53; 95% CI 1.14–2.06). In a stratified analyses by age, sex, and ethnicity, melanoma was observed to predict the diagnosis of BP in patients older 80 years of age (OR 1.63; 95% CI 1.11–2.38), males (OR 1.66; 95% CI 1.09–2.54), and individuals of Jewish ethnicity (OR 1.54; 95% CI 1.15–2.07; Table 2).

**Table 2:** The risk of bullous pemphigoid in patients with a preceding diagnosis of melanoma stratified by age, sex, and ethnicity (case–control study design)

Subgroup	Melanoma in patients with BP *n* (%)^a^	Melanoma in controls *n* (%)^a^	OR (95% CI)	Univariate *P* value
All	59 (1.5%)	190 (1.0%)	**1.53 (1.14–2.06)**	**0.004**
Age, years				
< 70	6 (0.7%)	33 (0.7%)	0.93 (0.39–2.23)	0.873
71–80	17 (1.5%)	51 (0.9%)	1.69 (0.97–2.93)	0.061
≥ 80	36 (1.9%)	106 (1.2%)	**1.63 (1.11–2.38)**	**0.012**
Sex				
Male	29 (1.8%)	86 (1.1%)	**1.66 (1.09–2.54)**	**0.018**
Female	30 (1.3%)	104 (0.9%)	1.43 (0.95–2.15)	0.087
Ethnicity				
Jews	59 (1.6%)	189 (1.0%)	**1.54 (1.15–2.07)**	**0.004**
Arabs	0 (0.0%)	1 (0.1%)	NA	0.658

*OR* odds ratio, *n* number, *CI* confidence interval

^a^The prevalence of melanoma in cases when melanoma preceded BP (in cases) or preceded recruitment (in controls)

Bold: significant value

We then performed two sensitivity analyses. The first of which by including only cases managed by BP-related medications and the second omitted cases and controls given a diagnosis of melanoma in the initial 2 years of the study. The association kept its statistical significance in both of these sensitivity analyses (OR 1.54; 95% CI 1.12–2.11 and OR 1.43; 95% CI 1.06–2.92, respectively).

The association was not meaningfully altered after adjusting for demographic variables (model 1; OR 1.52; 95% CI 1.13–2.05) as well as for demographic variables alongside comorbidities (model 2; OR 1.54; 95% CI 1.14–2.08) and additionally for exposure to PD-1/PDL-1 inhibitors, Parkinson’s disease, and overutilization of healthcare services (model 3; OR 1.45; 95% CI 1.07–1.96; Supplementary Table 1).

### The risk of developing melanoma among patients with bullous pemphigoid

The incidence rate of melanoma was 1.42 (95% CI 0.95–2.05) and 1.16 (95% CI 0.96–1.37) per 1000 person-years among patients with BP and controls, respectively (Table 3). Compared to control subjects, the risk of incident melanoma was not significantly elevated among patients with BP, neither in the unadjusted (HR 1.18; 95% CI 0.77–1.82; *P* = 0.447) nor in the adjusted (adjusted HR 1.13; 95% CI 0.73–1.74; *P* = 0.587) analyses. This finding was not altered after a sensitivity analysis which included only BP patients managed by BP-related medications (Table 3).

**Table 3:** Incidence rates and hazard ratio of new-onset melanoma among patients with bullous pemphigoid (cohort study design)

	Patients with bullous pemphigoid	Controls
Follow-up time, PY	18,320.9	106,465.8
Median follow-up time, years (range)	3.49 (0.00–17.64)	4.66 (0.00–17.85)
Number of events of melanoma	26	123
Incidence rate/1000 PY	1.42	1.16
95% CI	0.95–2.05	0.96–1.37

	HR (95% CI)	*P* value
Crude	1.18 (0.77–1.82)	0.447
Adjusted^a^	1.13 (0.73–1.74)	0.587
Sensitivity analysis^b^		
Crude	1.07 (0.69–1.66)	0.768
Adjusted^a^	1.06 (0.67–1.65)	0.809

*HR* hazard ratio, *CI* confidence interval, *PY* person-year

^a^Following the adjustment for age, sex, ethnicity, socioeconomic status, comorbidities, PD-1/PDL-1 exposure, healthcare utilization, and Parkinson diseases

^b^Sensitivity analysis included only bullous pemphigoid patients under prolonged “bullous pemphigoid-related treatments”

Bold: significant value

### The time sequence of the appearance of the two investigated conditions

Of the 85 patients with a dual diagnosis of BP and melanoma, BP followed melanoma in 59 (69.4%) cases whilst it preceded melanoma in the remaining 26 (30.6%) cases. When BP followed melanoma, the mean (SD) interval between the conditions was 9.2 (7.7) years, and the median (range) interval was 7.8 (0.1–36.0) years. When BP preceded the onset of melanoma, the average (SD) and the median (range) intervals were 3.5 (2.6) and 2.9 (0.2–8.8) years, respectively.

### The epidemiological and clinical features of patients with BP and melanoma as compared to the remaining patients with BP

Patients with coexistent BP and melanoma were significantly older at the onset of BP (80.4[10.2] vs. 76.6[14.4]; *P* = 0.001), had lower body mass index (25.8 [3.5] vs. 27.9 [6.1]; *P* < 0.001), and higher exposure to PD-1/PDL-1 inhibitors (1.2% vs. 0.1%; *P* = 0.004).

**Fig. 1:**
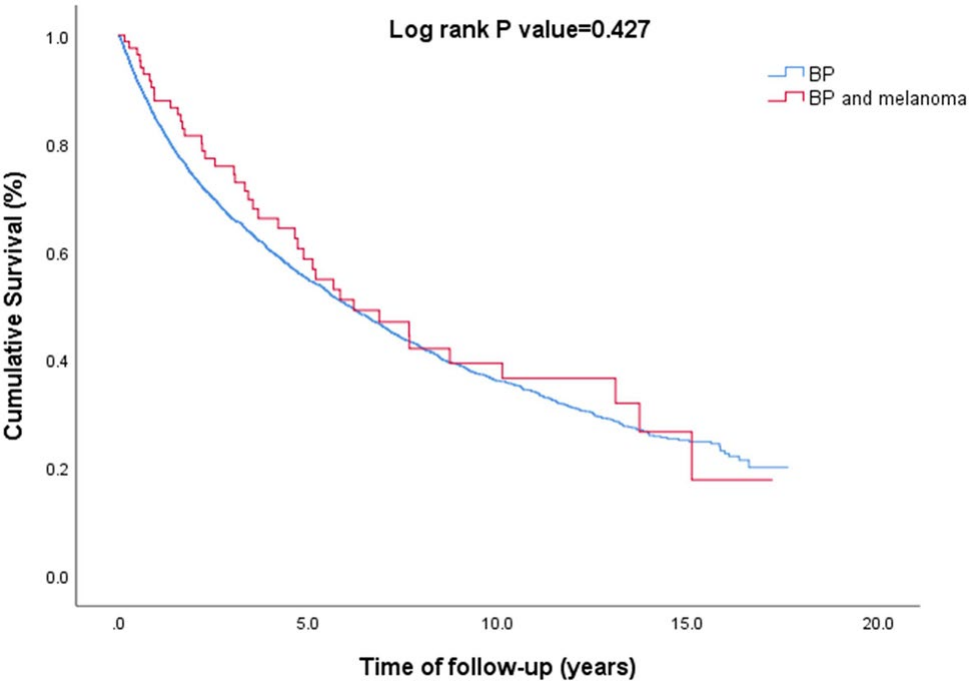
A Kaplan–Meier curves demonstrating survival of patients with BP and comorbid melanoma relative to the remaining patients with BP

The two subgroups were comparable with respect to sex, ethnicity, Charlson comorbidity index, exposure to long-term systemic corticosteroids and adjuvant therapies, and the prevalence of smoking and the recently reported dipeptidyl peptidase 4 inhibitors (DPP4i)-associated BP (Table 4). The risk of all-cause mortality was comparable between patients with BP and comorbid melanoma as compared to those with isolated BP (HR 0.88; 95% CI 0.65–1.20; *P* = 0.427; Fig. 1).

**Table 4:** Comparison between patients with coexistent bullous pemphigoid and melanoma relative to the remaining patients with bullous pemphigoid

	BP with melanoma (*n* = 85)	BP without melanoma (*n* = 3839)	*P* value
Age at the onset of BP, years; mean (SD)	80.4 (10.2)	76.6 (14.4)	**0.001**
Male sex, *n* (%)	44 (51.8%)	1623 (42.3%)	0.079
Jewish ethnicity, *n* (%)	84 (98.8%)	3668 (95.5%)	0.144
Body mass index; kg/m^2^, mean (SD)	25.8 (3.5)	27.9 (6.1)	** < 0.001**
Smoking, *n* (%)	26 (30.6%)	1122 (29.2%)	0.779
Charlson comorbidity Index (w/o malignancies); mean (SD)	2.7 (1.8)	2.9 (2.2)	0.413
Long-term systemic corticosteroids, *n* (%)^a^	58 (68.2%)	2494 (65.0%)	0.540
Long-term topical corticosteroids, *n* (%)^b^	83 (97.6%)	3609 (94.0%)	0.164
Adjuvant immunosuppressant or immunomodulatory agents^c^	54 (63.5%)	2283 (59.5%)	0.457
DPP4i-associated BP, *n* (%)	5 (5.9%)	291 (7.6%)	0.558
PD-1/PDL-1 antagonists-associated BP, *n* (%)	1 (1.2%)	5 (0.1%)	**0.004**

*n* number, *SD* standard deviation, *w/o* without, *DPP4i* dipeptidyl peptidase-4 inhibitor, *PD* programmed death, *PDL* programmed death ligand

Bold: significant values

^a^Patients managed by systemic corticosteroids for more than 6 months

^b^Patients managed by topical corticosteroids for more than 6 months

^c^Patients managed by one of the following agents: azathioprine, mycophenolate mofetil, methotrexate, cyclophosphamide, dapsone, doxycycline, rituximab, plasmapheresis, intravenous immunoglobulins

## Discussion

The current large-scale population-based study revealed that patients with a preexisting diagnosis of melanoma are at a 1.5-fold increased risk of developing BP. This association was most prominent among males and individuals older than 80 years of age and persisted following the adjustment for putative confounding factors, including exposure to PD-1/PDL-1 antagonists. Compared to the remaining patients with BP, those with BP and comorbid melanoma were older at the onset of BP and had lower BMI.

The association of BP with melanoma is poorly understood, as it is based merely on case reports. In 2017, Amber et al. [10] summarized the literature to reveal five cases of BP developing in association with melanoma. Intriguingly, the clinical course of BP paralleled that of melanoma in those reported patients, with several patients experiencing flares in BP in association with the development of metastatic diseases [19–21]. Correspondingly, patients with BP achieved a notable improvement in their cutaneous disease following the resection of the initial melanoma or the metastatic lymph nodes [10, 19].

A growing body of evidence accumulated in the past few years to suggest an association between administrations of PD-1/PDL-1 antagonists, part of which utilized to manage patients with metastatic melanoma, and the development of subsequent BP [16]. PD-1 and PDL-1 normally function as immune checkpoints which prevent the activation of T-cells and promote self-tolerance. The PD-1/PD-L1 pathway represents an adaptive immune resistance mechanism that is utilized by tumors to escape immune surveillance [22]. PD-1/PDL-1 directed monoclonal antibodies were recently developed and demonstrated promising anti-cancer activity [23], but were strongly associated with immune-related adverse events, including the induction of BP, which was proved morphologically and immunopathologically indistinguishable from typical BP [16]. Of great interest, the increased risk of BP among individuals with a history of melanoma persisted following the adjustment for these agents. This indicates that melanoma embodies an independent risk factor of BP regardless of exposure to PD-1/PDL-1 antagonists. It cannot be thoroughly refuted that the increased incidence of BP under PD-1/PDL-1 antagonists among metastatic melanoma patients actually represents an exacerbation of the underlying association between melanoma and BP.

The pathomechanism underlying the association between BP and melanoma is yet to be fully elucidated. However, several observations may lend weight to this epidemiological comorbidity. Krenacs et al. [11] found that the cell-residual 60 kDa endodomain of BP180 is expressed in malignant melanoma but not in benign melanocytic tumors, namely common, blue, and Spitz nevi. The authors postulated that the accumulation of BP180 endodomain in melanocytic tumors is associated with malignant transformation. In their recent study, Hwan et al. [12] generated a mouse strain with BP180 dysfunction and showed that it is associated with myeloid-derived suppressor cells (MDSC) influx into the skin as well as with melanoma progression. This study implies that BP180 exerts an antitumor effect through modulating the infiltration of MDSC. In a Japanese study, levels of anti-BP230 autoantibodies were significantly increased among 55 patients with melanoma relative to 27 healthy controls. Intriguingly, these autoantibodies were detected in melanoma patients at both early and advanced stages of disease [13]. A subsequent study comparing 179 patients with melanoma and 22 controls failed to find any significant differences, neither in seropositivity nor in levels of these autoantibodies [24]. The aforementioned studies throw light on a putative link between melanoma and the autoantigens of BP, which may contribute to understanding our epidemiological findings. Further research is required to better elucidate the mechanism of this comorbidity.

On the genetic level, human leukocyte antigen (HLA) polymorphisms might impose a predisposition for both BP and melanoma. HLA-DQB1*03:01 was found overrepresented in Caucasian patients with melanoma and independently predicted recurrence and metastasis [25, 26]. Likewise, HLA-DQB1*03:01 strongly interacts with BP180 and is the most established HLA allele to associate with BP [27, 28].

The current study represents the first population-based study aiming to investigate the association between melanoma and BP. Owing to the knowledge of the temporal sequence in which the diagnosis appeared, we were able to elucidate the risk of melanoma among patients with BP and the risk of subsequent BP in those with a history of melanoma [29]. The population-based setting enabled to include patients managed in all levels of healthcare facilities, thus arguing against the presence of selection bias. Being of a large-scale nature, the current study provides findings that are less likely to pop up due to chance. Since it is based on a computerized dataset, the current study lacked morphological and pathological data of the eligible patients (e.g., the stage of melanoma and severity of BP).

In conclusion, the current population-based study depicts that a history of melanoma places the patient at a 50% increased risk of developing subsequent melanoma. This risk was independent of the exposure to PD-1/PDL-1 antagonists and was robust to sensitivity and multivariate analyses. The current study provides the first epidemiological evidence of an association between BP and melanoma and substantiates previous scattered observations attributing a role for BP autoantigens in carcinogenesis of melanoma. Further experimental research is necessary to better delineate the mechanism underlying this association. Knowledge about this association may motivate physicians managing melanoma patents to avoid additional well-recognized risk factors of BP like exposure to DPP4i and neuropsychiatric medications.

## Supplementary Information

Below is the link to the electronic supplementary material.

Supplementary file1 (DOCX 14 KB)
