# Supplementary material for: Melanoma is associated with an increased risk of bullous pemphigoid: a large population-based longitudinal study
Source: Arch Dermatol Res. 2021 Mar 9;314(1):77–83. doi: 10.1007/s00403-021-02211-4 (PMC8741710; doi:10.1007/s00403-021-02211-4)
Supplement: Supplementary file 1 — Supplementary file1 (DOCX 14 KB) [file 403_2021_2211_MOESM1_ESM.docx]

**Supplementary Table 1:** Multiple logistic regression analysis of the association between melanoma and the later development of BP (case-control study design)

|  | **OR** | **95% CI** | **P value** |
| --- | --- | --- | --- |
| **Regression model** |  |  |  |
| Unadjusted model | 1.53 | 1.14-2.06 | **0.004** |
| Model 1^a^ | 1.52 | 1.13-2.05 | **0.005** |
| Model 2^b^ | 1.54 | 1.14-2.08 | **0.007** |
| Model 3^c^ | 1.45 | 1.07-1.96 | **0.016** |

^a^ adjusted for age, sex, ethnicity, and socioeconomic status

^b^ adjusted for age, sex, ethnicity, socioeconomic status, and comorbidities (as estimated by Charlson comorbidity index)

^b^ adjusted for age, sex, ethnicity, socioeconomic status, comorbidities (as estimated by Charlson comorbidity index), Parkinson disease and overutilization of healthcare system

**Abbreviations:** CI, confidence interval;

**Bold:** significant values
